# Human Health Risk Assessment by Dietary Intake and Spatial Distribution Pattern of Polybrominated Diphenyl Ethers and Dechloran Plus from Selected Cities of Pakistan

**DOI:** 10.3390/ijerph17249543

**Published:** 2020-12-20

**Authors:** Adeel Mahmood, Jabir Hussain Syed, Waseem Raza, Amtul Bari Tabinda, Andleeb Mehmood, Jun Li, Gan Zhang, Mudassar Azam

**Affiliations:** 1 Department of Environmental Sciences, Faculty of Natural Sciences, GC Women University, Sialkot 51310, Pakistan; adilqau5@gmail.com; 2 State Key Laboratory of Organic Geochemistry, Guangzhou Institute of Geochemistry, Chinese Academy of Sciences, Guangzhou 510640, China; junli@gig.ac.cn (J.L.); zhanggan@gig.ac.cn (G.Z.); 3 Department of Meteorology, COMSATS University, Islamabad Tarlai Kalan, Park Road Islamabad, Islamabad 45550, Pakistan; shahg14@gmail.com; 4 State Key Laboratory of Fine Chemicals, Dalian University of Technology, Dalian 116024, China; razawaseem2@yahoo.com (W.R.); andleeb.mehmood@gmail.com (A.M.); 5 Sustainable Development Study Centre, Government College University, Lahore 54000, Pakistan; amtulbaritabinda@gcu.edu.pk; 6 Institute of Chemical, Environmental and Bio Science Engineering, TU Wien, 1060 Vienna, Austria

**Keywords:** PBDEs and DPs, cereal crops, human health hazards, ecological risks, dietary intake

## Abstract

A class of intractable bio accumulative halogenated compounds polybrominated diphenyl ethers (PBDEs) was studied. Specifically, PBDEs and dechloran plus (DP) contamination in wheat and the assaulted environment—agricultural soil and dust—from metropolitan cities of Pakistan was the focus. The exposure of brominated flame retardants (BFRs) to humans, their probable toxicological impact on health, source apportionment, and the spatial tendency of BFRs were studied. Chromatographic analysis was performed, and concentrations (ng g^−1^) of ΣPBDE and ΣDP in soil, dust, and cereal crops were estimated in a range from 0.63 to 31.70 n.d. to 6.32 and n.d. to 3.47, respectively, and 0.11 to 7.05, n.d. to 4.56 and 0.05 to 4.95, respectively. Data analysis of source apportionment reflected that the existence of solid and e-waste sites, long-range transport, urban and industrial fraction can be the potential source of PBDE and DP pollution. Moreover, potential hazardous risks to human health across the study area via the dietary intake of cereal foods were deemed trifling, and were gauged on the basis of existing toxicological data.

## 1. Introduction

Flame retardants (FRs) are used in various combustible tools to meet fire-safety regulations and minimize the threat of fire. About 175 chemical compounds are known that exhibit FR properties and they are divided into four categories: halogenated organic, inorganic, phosphorus, and nitrogen-containing compounds [1,2,3]. Easily accessible brominated FRs are the members of halogenated organic compounds, which are broadly used as FRs in a variety of materials such as textiles, plastics, furniture, foam, electric household equipment, and electronic goods. Furthermore, 38% of the total global bromine is used in brominated flame retardants (BFRs) [4,5]. Additive BFRs are associated with polymers and are more likely to be released into the environment because of their ability to easily percolate out of products as compared to reactive BFR counterparts. Polybrominated diphenyl ethers (PBDEs) are an example of additive FRs, used widely among other FRs [6,7]. Structurally, PBDEs are like polychlorinated biphenyls (PCBs), with the major difference being that the presence of a bromine substitute lies in the presence of the ether group, linking the two phenyl rings. Among PBDEs’ technical mixtures, penta-, octa-, and deca-BDE are commonly used in the industry [8,9]. For example, penta-BDE is used in foam, while octa-BDE is an essential constituent of high-impact polystyrene. The rigid-plastic-item, textile, resin, pigment, and plastics industries extensively use the deca-BDE formulation [6]. These are persistent organic pollutants that stay in the environment for a long time (more than a year) without degradation. Among highly bioaccumulative pollutants, brominated congers are remarkable. PBDEs may cause a spectrum of chronic diseases from cognitive disorders to hormonal and liver dysfunction. Dechloran plus (DPs) are another highly chlorinated flame-retardant group that has been used for decades in connector coating, cables, and electric wires [10]. DPs received much attention when they were observed in sediments, soil, and food commodities in Pakistan [7,11].

In the 1970s, PBDEs were detected in the environment for the first time. By the 1990s, concern regarding the occurrence of PBDEs in the environment had increased among scientists and policymakers [12,13]. Sediment core samples from the Baltic Sea showed an exponential updrift in PBDEs since the 1970s [9,14]. In contrast, PCBs, DDTs (Dichloro-diphenyl-trichloroetahne), and dioxins in the same core sample exhibited a decreasing trend. PBDEs are of global concern and considered universal pollutants, as they were reported from remote areas like the Arctic, Antarctica, and deep oceans [15,16,17,18]. There is scarcity of data on the occurrence and effects of PBDEs on food commodities, animals, and human wellbeing [19].

Limited data were published from Pakistan on PBDE and DP contamination in food crops and the environment, and they revealed the existence of this contaminant group in the country [5,6,7,9,11,20]. The current study was designed to investigate PBDE and DP contamination in Pakistani food crops and the environment. Exposure to humans and animals, their probable toxicological impact on human life, source apportionment, and distribution pattern across the Grand Trunk (GT) Road from Lahore to Peshawar (Pakistan) were investigated. Results provide needed information to examine FR exposure pathways and point sources in the study area.

## 2. Material and Methods

### 2.1. Study Area and Sampling Strategy

**Figure 1:**
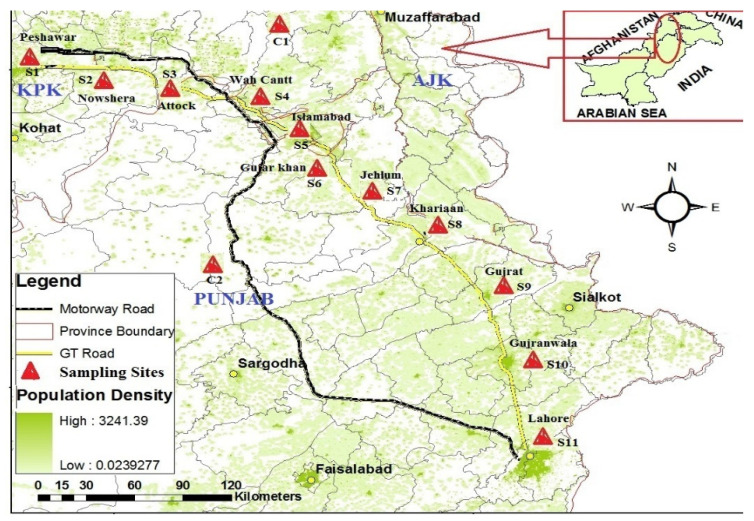
Map of study area showing Grand Trunk (GT) Road covered in this study.

The agricultural belt (approximately 512 km) and the GT road from Peshawar to Lahore were selected as the study areas, which lie between 31°32′–34°02′ N to 74°22′–71°37′ E, shown in Figure 1. The study area consists of two zones based upon land-use type and province boundary, namely, Khyber Pakhtunkhwa (KPK) and Punjab (PNJ). From the KPK zone, three different sites, S1, S2, and S3, were selected for the collection of wheat grains, agricultural soil, and surrounding roadside dust samples, while eight sites were selected from the PNJ zone for a sampling of a similar matrix. Two sites, C1 and C2 (facing the fewest anthropogenic activities), were selected from each zone of the allocated zones, as shown in Table S1 and Figure 1. Selection of respective sites from the allocated zone was grounded upon human activities along the GT road, such as variance in habitat, urbanization, and electronic and solid-waste discarding.

### 2.2. Field Sampling

#### 2.2.1. Agricultural-Soil and Wheat-Grain Sampling

A total of 52 composite samples of agricultural soil and wheat grains (*n* = 6 for each matrix) were collected from 13 sampling sites across the GT Road. Each site was used to collect two composite samples across the roadside and not far from the main urban settlement. Agricultural fields were limited across the urban settlements, which restricted the sampling size. Each sample was the composite of 4–5 subsamples that were properly mixed, dried, and sieved using a 2 mm sieve, and saved properly. Soil samples collected from the root of wheat plants were used to collect the wheat-grain samples. Samples were stored in a freezer until further investigation.

#### 2.2.2. Dust Sampling

Dust samples (*n* = 26) were collected from each site located across the GT Road. A foliar layer of dust on plant leaves and roadside items was carefully collected and placed in small bags. Composite samples were collected from each site and kept in polythene bags before being transported to the laboratory for storage and analysis.

#### 2.2.3. Extraction and Clean-Up Procedure

Soil, dust (10 g), and wheat (20 g) were Soxhlet-extracted by dichloromethane (DCM) for 24 h. Decachlorobiphenyl (PCB-209) as the surrogate standard mixture was added in the samples prior to extraction. Elemental sulfur was removed by activated granules of copper from dust and soil samples. The final fraction was concentrated to 0.2 mL under a gentle high-purity nitrogen stream after adding 25 μL of dodecane as a solvent keeper [3]. Prior to gas chromatography–mass spectrometry (GC-MS) analysis, a known quantity of BDE-77 was added as an internal standard. A flowchart for experimentation is provided in Figure S1.

### 2.3. Chromatographic Analysis

Eight BDE congeners (28, 35, 47, 99, 100, 153, 154, and 183) were determined by GC–MS equipment with a DB5-MS capillary column (30 m × 0.25 mm i.d.; 0.25 µm film thickness). Temperature was set to 150 °C for quadruple and source method detection limits (MDLs). Total run time was no more than 20 min with vacuum compensation. He was the used carrier gas [21].

### 2.4. Quality Control and Quality Assurance (QC/QA)

Samples were pretreated by following a strict quality-control (QC) procedure to ensure the merit of results. Analytical-grade chemicals were used for experimentation, purchased from Merck (Germany). Surrogate recovery, internal and other standards used for BDEs and DPs were purchased from Germany (Dr. Ehrenstorpher GmbH). Field, procedural, and solvent blanks were screened by adopting a similar methodology as that used for the original samples. Glassware was double-washed and baked at 450 °C for more than 5 h. Data processing and acquirement were assessed by Chemstation software (Agilent MSD Productivity Chemstation). USEPA method 5055 was followed to calculate MDLs. Calculated MDLs for PBDEs in soil, wheat, and rice samples ranged from 2 to 6 pg. g^−1^ [5]. Average surrogate recoveries of TCmX and PCB-209 for all detected samples ranged from 53% to 68%, and 77% to 81%, respectively.

## 3. Results and Discussion

### 3.1. PBDE and DP Profile

#### 3.1.1. Agricultural Soil

A summary of basic descriptive data of BDE and DP levels in the soil collected from agricultural fields located along GT Road is presented in Table 1. Concentrations (ng g^−1^) of ΣPBDE ranged from 0.63 to 31.7 ng g^−1^ with a mean concentration of 10.10 ± 8.76, while ΣDP levels were found in the range of 0.11–7.05 (average: 1.09 ± 1.87). Among individual congeners, BDE-47 was found predominant, over others with 32%, followed by BDE-100, BDE-99, BDE-154, BDE-153, BDE-83, BDE-28, and BDE-35, while *syn*-DP dominated over *anti*-DP. Penta-BDE was the predominant homolog by contributing 36% of total BDEs, followed by tetra- and deca-BDE.

The currently reported concentration of PBDEs was found to be lower than that of a published report from Pakistan (21.1 ± 8.1 ng g^−1^) and previously published data from Pakistan (40 ± 98 ng g^−1^) [6,11]. Our results exhibited higher levels of PBDEs as compared to those of previously published reports from the Yangtze River Delta, China (mean, 0.76 ng g^−1^) and the central Loess Plateau, China (0.035–12.1 ng g^−1^) [22]. These results were lower than those published for soil samples collected from e-waste burning sites of Turkey (0.05–2840 ng g^−1^); Norway, United Kingdom; and southern China (77–249 ng g^−1^) [23,24,25]. Global data on DP levels are limited, and the discussion and comparison in this study were prepared on the basis of the available reports. Our results depicted higher levels of DP as compared to those of a previously published report along River Chenab tributaries (0.64 ± 0.6 ng g^−1^) and selected districts of Punjab Province (0.80 ± 2.10 ng g^−1^) [6,11]. When a comparison was made with other global regions, results of the current report were found to be lower than those in Harbin, northeastern China (11 ng g^−1^), industrial areas in China (0.034–4.65 ng g^−1^), and a DP production facility in Huai’an, China (0.59–6.27 ng g^−1^) [26,27,28].

#### 3.1.2. Dust

Dust samples were analyzed for PBDE and DP investigation, which revealed that, similar to the soil samples, PBDE-47 was predominant over other PBDE congeners by a 33% contribution to total PBDEs. Ranks for BDEs by concentration levels were, in descending order: BDE-100 = BDE-99 > BDE-153 > BDE-83 = BDE-28 = BDE-35 > BDE-154. Results revealed that *syn*-DP was predominant over *anti*-DP, while concentrations of *fanti* were also found to be considerable. Details about basic descriptive statistical values of individual congeners of PBDEs and DP are provided in Table 1. Results of homolog patterns reflected that tetra-BDEs were the highest, followed by penta-, tri-, and deca-BDEs. In detail, homolog patterns for dust samples were similar to the homolog pattern reported for soil in this study, and it was observed to be in accordance with a previously reported study from Pakistan [11,29].

Results of this report were compared with previously published data on PBDEs for dust samples across different countries. Our results were comparable with data reported from Belgium, and lower than those in the UK, Japan, Australia, Germany, and Sweden [30,31,32,33,34,35].

#### 3.1.3. Cereal Crop (Wheat)

Summarized basic descriptive statistical values of PBDEs and DP investigated from wheat-grain samples are shown in Table 1. Overall, concentrations (ng g^−1^) of ∑BDEs and ∑DP ranged from n.d. to 3.47 (average: 0.49 ± 0.95) and 0.05 to 4.95 (average: 0.75 ± 1.32). Levels of DP in wheat samples were found to be higher than BDE level weres. The European Food Safety Authority published a similar profile for BDEs on foodstuffs; however, no specific report such as this one on cereal crops is available [36]. In this study, BDE-47 was the predominant congener with 26%, followed by BDE-99, BDE-35, BDE-28, BDE-153, BDE-183, and BDE-154, while *syn*-DP dominated over *anti*-DP. This reported trend of BDE levels was similar to a previously reported profile of BDEs from the United States and Pakistan, where BDE-47, 99, and 28 were the predominant congeners in food commodities [11,37]. The dominance of BDE-47, -99, and -28 is evidence that lower brominated PBDEs are more common in environmental compartments of the country and in assaulting the food-crop environment. Among BDE homologs, penta-BDEs contributed 40% of total PBDEs, while tetra-BDEs were 26%, tri-BDEs were 18%, and deca-BDEs contributed 16% of PBDEs.

There is a global scarcity of published data for PBDEs and DPs in cereal crops; our comparison was made with available published data of detected PBDE and DP levels in food crops. Our results were similar to or considerably higher than those of reports published for cereal crops from Catalonia, Spain in 2000 (n.d to trace ng kg^−1^); rice and wheat products from Catalonia, Spain in 2006 (rice, 31.1; wheat, 42.5 ng kg^−1^); vegetables from Japan (38.4–134.0 ng kg^−1^); and food commodities from the United Kingdom (90 ng day^−1^), Canada (44 ng day^−1^), and America (12–16 pg. g^−1^) [37,38,39,40,41,42]. However, Roszko et al. reported higher levels of wheat (1.11 ng g^−1^) collected from Poland [43]. DP levels reported in our study were found to be lower than those in previously published studies (0.45–16.6 ng g^−1^) from China [44]. Plant samples were collected from an e-waste burning site of China, which might be the reason for the higher levels of reported DP from China.

**Figure 2:**
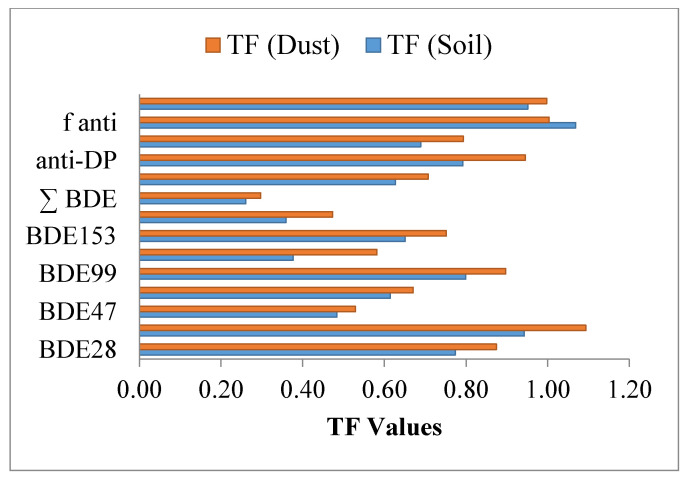
Transfer-factor (TF) values of soil (red) and dust (blue) into wheat for bioaccumulation of target compounds (from BDE28 to f anti).

Results of the bioaccumulation pattern of PBDEs and DP in wheat grains from dust and soil revealed that dust has more influence on detected levels of target compounds in wheat as compared to the soil, as shown in Table 2 and Figure 2. This may be due to the locality of sampling sites across the heavily mobilized GT Road, and in the vicinity of urban and industrial areas.

### 3.2. Spatial Distribution and Source Apportionment

**Figure 3:**
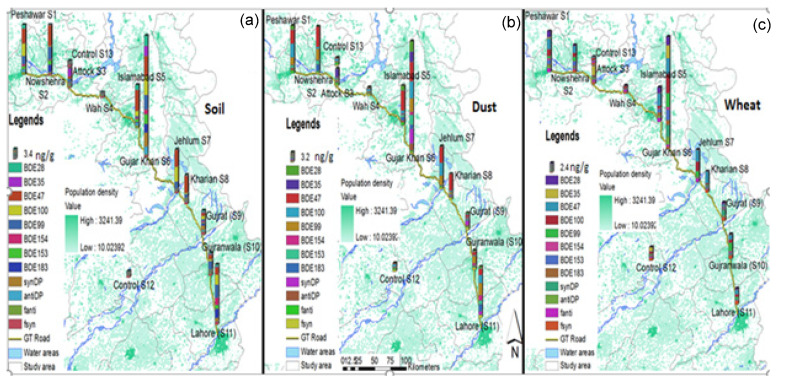
Spatial distribution pattern of PBDEs and DP along each site of the study area for (**a**) soil, (**b**) dust, and (**c**) wheat.

A summarized expression of the spatial distribution of PBDEs and DP across the study area is presented in Figure 3. Results of one-way ANOVA and the spatial-distribution pattern revealed a significant difference in detected contaminant levels among study sites across the GT Road. Samples were collected from hot spots of metropolitan cities facing high urban and industrial load. Samples collected from Gujar Khan were found to be highly contaminated with PBDEs and DP, perhaps due to the sampling-site selection. Industrial estate areas, automobile workshops, and electric-equipment shops, along with electric repair units, exist along the main GT Road, which passes across Gujar Khan. Figure S2 shows that Lahore is second with regard to contamination load, followed by Peshawar and Nowshera. Lahore and Peshawar are the capital cities of the Punjab and Khyber Pakhtunkhwa provinces of Pakistan, and renowned for facing high pollution load due to a huge setup of unplanned industrial units [45,46]. Agricultural land around the Ittehad chemical factories of Lahore, located along the GT Road and Ravi River, was selected for sample collection and seems to contribute to high contamination load. Similar topographic conditions were considered for sample collections from Peshawar, while Amangarh, Nowshera was selected for sample collection, as shown in Figure 1. The least contamination load of PBDEs and DP was observed at the S4 (Wah), S13, and S14 control sites, and reported levels (pg.) from these areas were negligible. These comparative results reflected that urban and industrial fractions, along with mobilization on the GT Road, played a vital role for PBDE and DP levels. In previously published reports from Pakistan, similar trends were reported, which reflects the urban, industrial, and mobilization impact on detected levels of pollutants [11,20,29,46,47]. Compared with China, similar trends were reported from Guangzhou for PBDE in air samples [48,49]. Moreover, the reported global literature verified that urbanized and industrialized areas have higher contamination loads as compared to those of periurban and rural areas, or localities far from industries or urbanization [24].

PBDEs were reported to be used in electric equipment, computer components, furniture industries, polystyrene, textile industries, foam units, electric connectors, etc. [50]. DPs are emitted from e-waste recycling and DP manufacturing units [51,52]. Air transportation may also transmit PBDEs and DPs from point sources to the neighboring environment through a long-range-transport (LRT) mechanism [10]. The usage, production, industrial application, and disposal/discharge of PBDE-associated products may be probable emission and contamination sources. Across the study area, few solid and e-waste disposal sites were detected (Lahore, Gujranwala, Gujar Khan, and Peshawar) along with the open burning of waste. Analytical results of this report reflected that the existence of solid and e-waste sites, LRT, an urban and industrial fractions may be substantial sources of PBDE and DP pollution.

### 3.3. Dietary Intake

**Figure 4:**
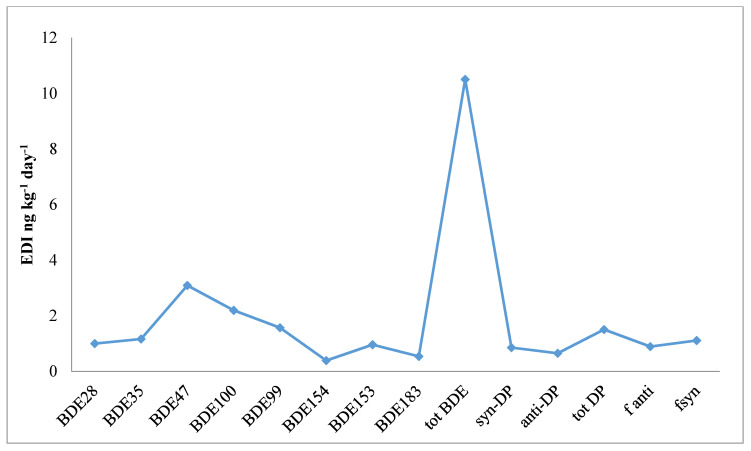
Calculated estimated daily intake (EDI) in ng kg^−1^ day^−1^ for wheat.

There is a global scarcity of published data for PBDE and DP concentrations from food commodities and their probable hazardous impact on human wellbeing. Estimated daily intakes (EDIs) of investigated compounds were calculated using average levels of PBDEs and DP from rice, followed by the EDI [5,43] for wheat consumption ranging from 0.4 to 2.2 ng kg^−1^ day^−1^ with an average of 1.9 ± 2.6 ng kg^−1^ day^−1^, as shown in Figure 4. Current results were compared with previously reported global EDI levels and were found to be higher than those of the adult population of Catalonia, Spain in 2003 and 2007 (740 and 630 pg. kg^−1^ day^−1^, respectively) by consumption of mixed cereal foods [41,53]. It is difficult to find published data on the EDI of cereal food crops, which made it difficult to compare the current report with diversified data. Published reports of PBDEs for mixed food commodities (seafood, fish, animal-originated food, etc.) provided very limited information on EDI [42,54,55,56,57,58]. Furthermore, BDE-28, -99, and -209 were recommended by the European Food Safety Authority (EFSA) for monitoring the plan in food commodities/routine of dietary foods [36]. During analysis of these samples, our instrument was not compatible for BDE-209 analysis, which created some complexity during the calculation of EDI and health risk assessment. When we looked at insight for the toxicological impact of the aforementioned compounds, a scrubby pattern of data appeared, and suggested that this area needs future focus.

Results of the current EDI report were found to be considerably higher than those of a published report from Pakistan (wheat; 0.002–0.035 pg. kg^−1^ day^−1^ and rice; 0.033–0.680 pg. kg^−1^ day^−1^) [11,29]. This difference may be due to the influence of the urban fraction on sampling locations of the study area, as in previously published reports, samples were collected from urban and periurban, and rural areas, while in this report, samples were collected purely from urban and industrial areas [45].

### 3.4. Probable Health Risks by PBDEs and DPs

Several national and international organizations developed protocols to evaluate and assess the probable risk to human health through PBDE and DP exposure by the intake of food commodities. Risk assessment was calculated through the acceptable daily intake (ADI) established by the WHO, minimal risk level (MRL) recommended by the Agency of Toxic Substances and Disease Registry (ASTDR), and hazard risk assessment by calculating cancer benchmark concentrations [59,60,61,62]. However, there is still a challenge to screen and assess potential risks to human health through the dietary intake of PBDEs and DPs, as toxicological and epidemiological data linked to these compounds are exceptionally sparse.

The lowest observed adverse effect level (LOAEL) of 1 mg kg^−1^ day^−1^ depends upon the sensitive lethal point of hazardous/toxic effects of PBDEs [41,62]. ASTDR provided the MRL for lower brominated PBDEs as 0.07 mg kg^−1^ day^−1^ and deca-BDEs as 10 mg kg^−1^ day^−1^ (http://www.atsdr. cdc.gov/mrls.html). In this report, the EDI was found to be lower than the suggested LOAEL and MRLs. Keeping in line with the guidelines, it is suggested that potential risks to human health across the study area through the dietary intake of wheat are trifling, assessed on the basis of available toxicological data. In our current study, the risk assessment of hazard impact on human health and EDI was calculated on the basis of detected residual levels of PBDEs and DP from wheat, dust, and their interacting environment.

## 4. Conclusions

Limited epidemiological and toxicological data of human health linked to PBDEs and DP resulted in difficulty in assessing actual human health hazards posed by these compounds. Long-range adverse health effects of these contaminants in the food chain, dust, and soil are emerging. Our report attempted to calculate the risk factor to human health by using available epidemiological data, and screening the levels and spatial distribution along the GT Road of Pakistan. Across the study area, few solid-waste disposal sites were detected, along with community settlements and agricultural land (Lahore, Gujranwala, Gujar Khan, and Peshawar), with the open and uncontrolled burning of waste. Analytical results reflected that the existence of solid and e-waste sites, long-range transport, and urban and industrial fractions might be potential sources of PBDE and DPs pollution. Congener-specific analysis revealed that BDE-47, BDE-99, and BDE-100 were predominant, whereas *syn-*DP was found to be dominant over *anti-*DP. EDI was lower than the suggested LOAEL and MRLs. Results suggested that potential risks to human health across the study area through the dietary intake of wheat were marginal, evaluated on the basis of available toxicological data. At this time, no regulatory efforts are being considered in Pakistan. It is recommended to stop the cultivation of food crops across contaminated waste sites and wastewater irrigation to food crops. More clinical and translational investigations are required in this area. Our results strengthen baseline data related to human health risk assessment of PBDEs and DP through food crops and the environment.

## Figures and Tables

**Table 1:** Descriptive statistical values in ng g^−1^ of polybrominated diphenyl ethers (PBDEs) and dechloran plus (DPs) for soil, dust, and wheat.

Compounds	Soil (ng g^−1^)				Dust (ng g^−1^)				Wheat (ng g^−1^)			
	Min	Max	Mean	St.Dev	Min	Max	Mean	St.Dev	Min	Max	Mean	St.Dev
BDE28	0.04	3.28	0.65	0.93	0.03	3.17	0.57	0.91	0.02	2.13	0.50	0.71
BDE35	0.01	3.44	0.62	1.08	0.01	3.32	0.53	1.06	0.00	3.26	0.58	1.05
BDE47	0.12	6.72	3.20	2.77	0.25	6.32	2.92	2.63	0.08	3.94	1.55	1.38
BDE100	0.07	6.22	1.79	2.12	0.17	5.37	1.64	1.78	0.12	4.75	1.10	1.25
BDE99	0.12	4.67	1.85	1.68	0.12	4.27	1.65	1.60	0.00	6.80	1.26	2.00
BDE154	0.05	2.33	0.51	0.69	0.06	1.58	0.33	0.44	0.02	0.75	0.19	0.21
BDE153	0.04	3.16	0.74	0.86	0.00	3.04	0.64	0.84	0.01	2.54	0.48	0.71
BDE183	0.04	2.86	0.75	1.12	0.02	2.19	0.57	0.78	0.01	1.15	0.27	0.34
∑BDE	0.63	31.70	10.10	8.76	1.51	27.54	8.85	7.54	0.00	3.47	0.49	0.95
*syn*-DP	0.03	4.66	0.68	1.27	0.00	4.56	0.60	1.26	0.02	2.99	0.43	0.84
*anti-*DP	0.04	2.39	0.41	0.64	0.01	2.30	0.34	0.63	0.00	1.96	0.32	0.54
∑DP	0.11	7.05	1.09	1.87	0.01	6.86	0.95	1.85	0.05	4.95	0.75	1.32
*fanti*	0.09	0.96	0.42	0.25	0.03	0.95	0.44	0.25	0.02	0.96	0.45	0.31
*fsyn*	0.04	0.91	0.58	0.25	0.05	0.97	0.56	0.25	0.04	0.98	0.55	0.31

**Table 2:** Bioaccumulation of PBDEs and DP from soil and dust to wheat. TF is transfer factor of soil and dust.

PBDE	BDE-28	BDE-35	BDE-47	BDE-100	BDE-99	BDE-154	BDE-153	BDE-183	∑BDE	syn-DP	anti-DP	∑DP	f*_anti_*	f*_syn_*
TF (Soil)	0.77	0.94	0.48	0.61	0.80	0.38	0.65	0.36	0.26	0.63	0.79	0.69	1.07	0.95
TF (Dust)	0.87	1.09	0.53	0.67	0.90	0.58	0.75	0.47	0.30	0.71	0.95	0.79	1.00	1.00

## Supplementary Materials

The following are available online at https://www.mdpi.com/1660-4601/17/24/9543/s1: Figure S1: Flowchart for experimentation of this study. Figure S2: Overall distribution and levels of detected congener along each site of study area. Table S1: Detailed description of study area.
