# Supplementary material for: Human Health Risk Assessment by Dietary Intake and Spatial Distribution Pattern of Polybrominated Diphenyl Ethers and Dechloran Plus from Selected Cities of Pakistan
Source: Int J Environ Res Public Health. 2020 Dec 20;17(24):9543. doi: 10.3390/ijerph17249543 (PMC7766582; doi:10.3390/ijerph17249543)
Supplement: Supplementary file 1 [file ijerph-17-09543-s001.pdf]

International Journal of
Environmental Research and Public Health

MDPI

Supplementary Material

Human Health Risk Assessment by Dietary Intake and Spatial Distribution Pattern of Polybrominated Diphenyl Ethers and Dechloran Plus from selected cities of Pakistan

Adeel Mahmood [1,5], Jabir Hussain Syed [2], Waseem Raza [3], Amtul Bari Tabinda [4], Andleeb Mehmood [3], Jun Li [5], Gan Zhang [5] and Mudassar Azam [6,*]

[1] Department of Environmental Sciences, Faculty of Natural Sciences, GC Women University, Sialkot and Pakistan;
[2] Department of Meteorology, COMSATS University, Islamabad Tarlai Kalan, Park Road Islamabad 45550 Pakistan and
[3] State Key Laboratory of Fine Chemicals, Dalian University of Technology, 116024, China;
[4] Sustainable Development Study Centre, Government College University, Lahore, Pakistan;
[5] State Key Laboratory of Organic Geochemistry, Guangzhou Institute of Geochemistry, Chinese Academy of Sciences, Guangzhou 510640 and China;
[6] Institute of Chemical, Environmental and Bio Science Engineering, TU WIEN. Austria
Corresponding Author: mudassar.azam@tuwien.ac.at

Table 1. Study area to demonstrate the samples and locations with the reference of map in Figure 1.

	Types of samples	Sampling area	Sampling sites
1.	Agricultural area soil	KPK	S1, S2 and S3
2.	Dust	Punjab	S4, S5, S6, S7, S8, S9, S10 and S11
**3.**	Cereal crop (wheat)	Control sites in Punjab and KPK	C1 (S13) and C2 (S12)

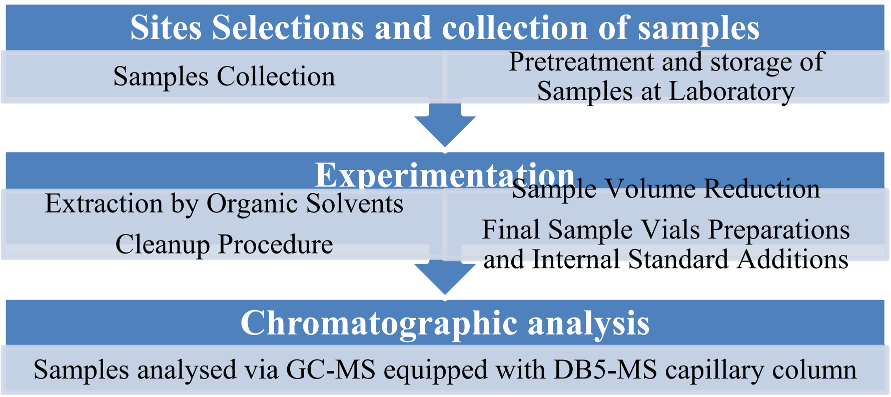

Figure S1. Flowchart showing the Sample preparations, cleanup, extraction and analysis procedure

International Journal of
Environmental Research and Public Health

MDPI

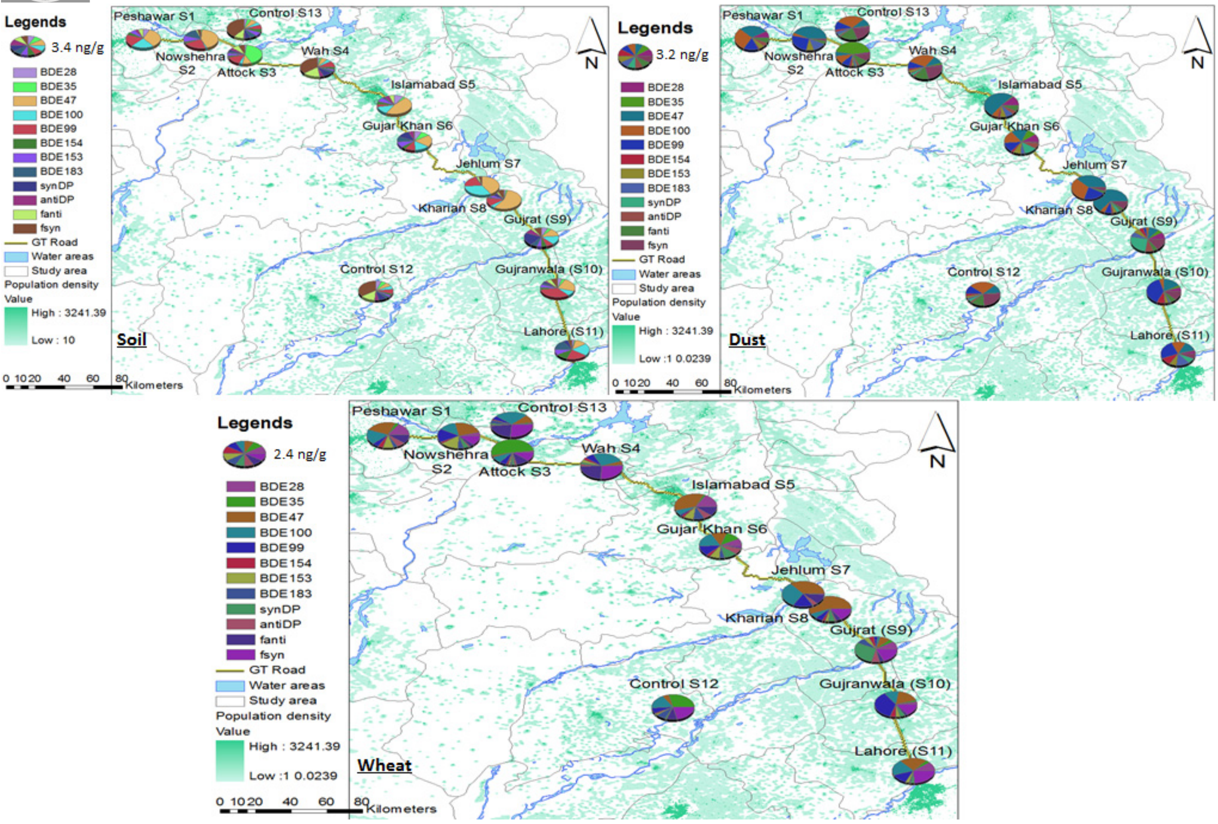

Figure S2. Overall distribution and levels of each detected congener along each site of the study area
